# Supplementary material for: Resolving the full spectrum of human genome variation using Linked-Reads
Source: Genome Res. 2019 Apr;29(4):635–45. doi: 10.1101/gr.234443.118 (PMC6442396; doi:10.1101/gr.234443.118)
Supplement: Supplemental Material [file supp_gr.234443.118_Supplemental_longranger-2.2.zip › longranger-2.2.tar.bz2!/lr2.2-public/lib/stan/cmdstan/stan/doc/api/html/dom__err_8hpp_source.html]

Stan: src/stan/math/error\_handling/dom\_err.hpp Source File


|  |  |
| --- | --- |
|  | Stan  2.5.0  probability, sampling & optimization |


- Main Page
- Related Pages
- Namespaces
- Classes
- Files

- File List
- File Members

All Classes Namespaces Files Functions Variables Typedefs Enumerations Enumerator Friends Macros Pages

- src
- stan
- math
- error\_handling

dom\_err.hpp

Go to the documentation of this file.

1 #ifndef STAN\_\_MATH\_\_ERROR\_HANDLING\_\_DOM\_ERR\_HPP

2 #define STAN\_\_MATH\_\_ERROR\_HANDLING\_\_DOM\_ERR\_HPP

3

4 #include <typeinfo>

5 #ifdef BOOST\_MSVC

6 # pragma warning(push) // Quiet warnings in boost/format.hpp

7 # pragma warning(disable: 4996) // \_SCL\_SECURE\_NO\_DEPRECATE

8 # pragma warning(disable: 4512) // assignment operator could not be generated.

9 // And warnings in error handling:

10 # pragma warning(disable: 4702) // unreachable code

11 // Note that this only occurs when the compiler can deduce code is unreachable,

12 // for example when policy macros are used to ignore errors rather than throw.

13 #endif

14 #include <boost/format.hpp>

15

16 #include <sstream>

17 #include <stdexcept>

18

19 namespace stan {

20  namespace math {

21

22  namespace {

23  // local output stream for pairs

24  template <typename T1, typename T2>

25  std::ostream& operator<<(std::ostream& o,

26  std::pair<T1,T2> xs) {

27  o << '(' << xs.first << ", " << xs.second << ')';

28  return o;

29  }

30  }

31

32  // currently ignoring T\_result

33  template <typename T,

34  typename T\_result,

35  typename T\_msg>

36  inline bool dom\_err(const char\* function,

37  const T& y,

38  const char\* name,

39  const char\* error\_msg,

40  const T\_msg error\_msg2,

41  T\_result\* result) {

42  std::ostringstream msg\_o;

43  msg\_o << name << error\_msg << error\_msg2;

44

45  std::string msg;

46  msg += (boost::format(function) % typeid(T).name()).str();

47  msg += ": ";

48  msg += msg\_o.str();

49

50  throw std::domain\_error((boost::format(msg) % y).str());

51

52  return false;

53  }

54

55

56  }

57 }

58 #endif

stan::math::dom\_err

bool dom\_err(const char \*function, const T &y, const char \*name, const char \*error\_msg, const T\_msg error\_msg2, T\_result \*result)

**Definition:** dom\_err.hpp:36

stan::gm::operator<<

std::ostream & operator<<(std::ostream &o, const expr\_type &et)

**Definition:** ast\_def.cpp:118


---

[ Stan Home Page ]

*© 2011–2014,
Stan Development Team.*
